# The Specific Encapsulation of Procyanidins from Litchi Peel and Coffee Pulp Extracts via Spray-Drying Using Green Polymers

**DOI:** 10.3390/polym15183823

**Published:** 2023-09-19

**Authors:** María de los Ángeles Vázquez-Núñez, Mayra Aguilar-Zárate, Ricardo Gómez-García, Carlos Reyes-Luna, Pedro Aguilar-Zárate, Mariela R. Michel

**Affiliations:** 1 Facultad de Estudios Profesionales Zona Huasteca, Universidad Autónoma de San Luis Potosí, Romualdo del Campo 501, Colonia Rafael Curiel, Ciudad Valles 79060, San Luis Potosí, Mexico; angeles.vazquezac@outlook.com; 2 Facultad de Ciencias Químicas-CIEP, Universidad Autónoma de San Luis Potosí, Av. Dr. Manuel Nava 6, Zona Universitaria, Mexico City 78210, San Luis Potosí, Mexico; mayra.aguilar@uaslp.mx; 3 CBQF—Centro de Biotecnologia e Química Fina—Laboratório Associado, Escola Superior de Biotecnologia, Universidade Católica Portuguesa, Rua Diogo Botelho 1327, 4169-005 Porto, Portugal; rgarcia@ucp.pt; 4 CIICYT—Centro de Investigación e Innovación Científica y Tecnológica, Unidad Camporredondo, Autonomous University of Coahuila, Saltillo 25280, Coahuila, Mexico; 5 Engineering Department, Tecnológico Nacional de Mexico/I. T. de Ciudad Valles, Carretera al Ingenio Plan de Ayala km 2, Colonia Vista Hermosa, Ciudad Valles 79010, San Luis Potosí, Mexico; carlos.reyes@tecvalles.mx

**Keywords:** polyphenols, whey protein, maltodextrin, Taguchi methodology, antioxidants, scanning electron microscopy

## Abstract

Polyphenols called procyanidins can be extracted from agro-industrial waste like litchi peel and coffee pulp. However, their efficacy is limited due to instability, which hinders both the bioavailability and preservation of their activity. This study aims to establish the ideal encapsulation conditions required to preserve the procyanidin properties found in extracts taken from litchi peel and coffee pulp. To attain the maximum procyanidin encapsulation efficacy (EE), the Taguchi method was utilized to streamline the spray-drying conditions for different wall materials—maltodextrin (MD), whey protein (WP), citrus pectin (CP), and skim milk (SM). The optimized conditions consisted of feed flow (3, 4.5, and 6 mL/min), temperature (125, 150, and 175 °C), and airflow (30, 35, and 40 m^3^/h). The microcapsules were characterized using ABTS, DPPH, lipoperoxidation, and scanning electron microscopy. Objective evaluations revealed that MD was the most effective encapsulation material for the litchi extract, whereas WP was the optimal option for the coffee extract. Of all the factors considered in the spray-drying process, feed flow had the strongest impact. The spray-drying process for the litchi peel extracts achieved high procyanidin encapsulation efficiencies at a feed flow rate of 4.5 mL/min, a temperature of 150 °C, and an airflow rate of 35 m^3^/h. Meanwhile, the coffee extract spray drying achieved similar results at a feed flow rate of 4.5 mL/min, a temperature of 175 °C, and an airflow rate of 40 m^3^/h. Encapsulation efficiencies of 98.1% and 93.6% were observed for the litchi and coffee extracts, respectively, under the mentioned optimal conditions. The microencapsulation process was successful in preserving the antioxidant properties of procyanidins. The microcapsules’ size ranged from 2.6 to 3.2 micrometers. The results imply that the phenolic compounds present in the extracts function as effective antioxidant agents.

## 1. Introduction

In recent years, a large number of active compounds from natural sources have been investigated. Polyphenols are the largest group of secondary metabolites found in plants [1]. They represent a large family of substances ranging from simple molecules to complex structures [2]. Polyphenols can modulate the activity of different enzymes and consequently interfere in signaling mechanisms and in different cellular processes, partly due to the physicochemical properties of these compounds. They also participate in various cellular metabolic reactions of oxidation–reduction [3].

A variety of polyphenolic compounds have been found in several edible plants. These compounds have been classified into different groups according to the number of phenolic rings and the structural elements connecting these rings [4]. Depending on the substitution pattern and the degree of oxidation that they possess, flavonoids can be classified into different groups [5]. The most representative types of flavanols in foods are of the flavan-3-ol type. These types of flavonols can be found as monomers (catechins) or as condensed dimers and oligomers (procyanidins), or they can appear as polymers (proanthocyanidins or condensed tannins) [6]. Procyanidins can be obtained from agro-industrial residues, such as litchi peel [2,7] and coffee pulp [7,8].

Recently, the research and application of polyphenols have been areas of great interest in the functional food industries [9] due to their biological activities, such as antioxidant, antimicrobial, anticancer, and other activities [10]. Unfortunately, their application is limited by the inherent instability. Furthermore, their effectiveness depends on the preservation of the bioavailability of the active ingredients, as well as the preservation of activity and controlled release. The process of encapsulation provides a compelling solution to these issues [11]. Rather than using free extracts, encapsulating procyanidins can effectively address instability while mitigating the unappealing smells and flavors associated with these compounds.

There are different techniques for the encapsulation of bioactive agents, and the main objective of all of them is to protect the core material from adverse environmental conditions, such as the undesirable effects of light, humidity, and oxygen, thus helping to increase the shelf life of the product [11,12]. Among the different techniques used for the encapsulation of bioactive compounds, spray drying is the most widely used technique in the food industry [13]. This method has many advantages, such as continuous operation, fast drying, and short exposure of the product to high temperatures [14].

Green polymers provide a compelling option as encapsulation matrices for bioactive molecules for several reasons. These polymers are abundant in nature and can even be sustainably sourced from natural or food-based sources, increasing their availability and providing nutritional value. Additionally, their cost-effectiveness makes advanced encapsulation technologies more accessible. These polymers have versatile chemical properties that enable tailored encapsulation approaches. Furthermore, their GRAS (Generally Recognized As Safe) status emphasizes their safety for various applications [15]. Examples such as maltodextrin, whey protein, pectin, and skim milk further highlight the potential of these green polymers in revolutionizing bioactive molecule encapsulation.

The present study aimed to identify the best conditions for encapsulating procyanidins extracted from litchi peels and coffee pulp. Different green polymers were tested in order to select the best matrices for encapsulating procyanidins. The Taguchi method was utilized to optimize the spray-drying process and attain the maximum encapsulation efficiency (EE), all while maintaining the bioactivities of the procyanidins.

## 2. Materials and Methods

### 2.1. Materials and Reagents

The matrices used were maltodextrin (JR Foods), citrus pectin (JR Foods), commercial skim milk (Svelty Nestle), and whey protein (Nature’s Best). All other chemicals used in the experimental procedures were acetone (Jalmek, NL, Mexico), ethanol (CTR, Monterrey, NL, Mexico), hydrochloric acid (Fermont, Monterrey NL, Mexico), Butanol (Karal, Mexico), ammonium iron (III) sulfate (Meyer, Mexico), formic acid (Jalmek, Mexico), Folin–Ciocalteu phenol (Sigma-Aldrich, USA), sodium carbonate (Jalmek, Mexico), ABTS (Sigma-Aldrich, USA), potassium persulfate (Meyer, Mexico), DPPH (Sigma-Aldrich, Germany), gallic acid (Sigma-Aldrich, USA), methanol (Jalmek, Mexico), acetate buffer (Fermont, Mexico), linoleic acid (Sigma-Aldrich, USA), Tween 20 (Hycel, Mexico), ferrous chloride (Jalmek, Mexico), EDTA (Hycel, Mexico), and sodium hydroxide (Jalmek, Mexico).

### 2.2. Plant Material

Litchi fruits were collected in the Tres Marias orchard (21.47612398510463 north, −98.97562266201969 west), in the municipality of Huichihuayan, S.L.P., Mexico, and coffee pulp was obtained from local cultivars in the municipality of Xilitla (21.3645260 north, −98.9632300 west), S.L.P., Mexico. The processing of the litchi fruits involved the separation of the shell from the pulp and seed, followed by drying the shell in a laboratory dryer (Memmert IN110) at 40 °C for 48 h. The coffee pulp was dried in a laboratory dryer (Memmert IN110) at 40 °C for 48 h. The resulting particles were separated using a mesh with openings of less than 1 mm.

### 2.3. Encapsulation Matrix Selection

#### 2.3.1. Extract Preparation

For the coffee pulp extract, 10 g of dried pulp was dispersed in 100 mL of an acetone–water solution at 70% (*w*/*w*). The preparation of the litchi extract was carried out by dispersing 10 g of dried husk in 100 mL of ethanol reagent (with 96% of purity). The extraction method was carried out utilizing the ultrasound-assisted method using Brason model 3800 equipment for 20 min at room temperature. Subsequently, the solutions were filtered, first with muslin fabric and then with filter paper. The obtained solutions were stored at a temperature of 4 °C in completely closed containers, isolated from light. All the extracts were obtained in triplicate.

#### 2.3.2. Quantification of Procyanidin Content in Litchi and Coffee Extracts

The content of procyanidins was quantified using the HCL–Butanol method [16] with some modifications. Hydrolysis was performed in 5 mL screw-capped glass tubes. The standard method of hydrolysis was as follows: 1.5 mL of a concentrated nBuOH-HCl solution (95:5 *v*/*v*), 50 µL of a 2% solution of ferric agent (prepared with NH_4_Fe(SO_4_)_2_•12H_2_O at HCI 2M), and 250 µL of the extract containing procyanidins were placed in tubes and thoroughly mixed in a vortex (Heathrow scientific, China). Then, they were placed in a water bath (Buchi, Heating Bath B-100) at a constant temperature of 95 ± 0.2 °C for 40 min. After this period, the tubes with the hydrolyzed procyanidins were removed from the bath and cooled at room temperature for 30 min. The next step was to analyze the hydrolyzed solution in a spectrophotometer (Genesys 10S UV-Vis Thermo Scientific) using an absorbance of λ = 550 nm.

#### 2.3.3. Microcapsules Preparation

For the preparation of microcapsules, four encapsulating materials were employed: maltodextrin (MD), skim milk (SM), whey protein (WP), and citrus pectin (PC). Initially, the solutions for the wall materials were made by dissolving 1% (*w*/*v*) of each material in 100 mL of distilled water under stirring conditions at room temperature. The solutions were subsequently stored for 24 h at 4 °C. After the storage period, 2 mL of each extract was dispersed in the corresponding encapsulating agent solution and left to stand for one hour. Then, microparticles loaded with the procyanidin-rich extract were produced with a theoretical extract loading of 0.2% (*w*/*w*) using a Mini Spray-Dryer B-290 (BÜCHI, Switzerland). The same procedure was repeated three times for each of the two extracts with each of the encapsulating materials.

#### 2.3.4. Release Technique

In microtubes, 10 mg of encapsulates was dissolved in 1 mL of a 5% (*v*/*v*) formic acid solution. They were thoroughly mixed in a vortex for 3 min and then centrifuged (Waverly centrifuge, model C200) at 8000 rpm for 5 min to precipitate the matrix remains. The resulting supernatant was collected for the procyanidin quantification procedure, which was performed for each encapsulate.

#### 2.3.5. Encapsulation Efficiency (EE)

After quantifying the content of procyanidins in each encapsulate, the encapsulation efficiency was calculated as follows:

(1) $$\%\;EE=\frac{CE}{CT}\times 100$$

where *CE* is the concentration of the procyanidins entrapped in the microcapsules, and *CT* is the concentration of the procyanidins existing in the extract. Three encapsulated materials were selected based on the encapsulation efficiency obtained for each extract in the various matrices.

### 2.4. Optimization of the Encapsulation Process

#### 2.4.1. Development of Taguchi Methodology

The Taguchi methodology allows one to choose a product or process that performs with greater consistency in the operating environment. A product designed with this goal in mind will perform more consistently, regardless of the environment in which it is used. In the present work, the Taguchi methodology was applied to optimize the spray-drying (Buchi Mini Spray Dryer B-290) process of the microcapsules. The effects of feed flow, air inlet temperature, and drying airflow were experimented with (Table 1). The orthogonal matrix design is shown in Table 2, where the orthogonal array L9 (3^3^) allowed one to develop the experimentation for the optimization of the microcapsule drying process.

#### 2.4.2. Encapsulation of the Extracts with the Selected Matrix

Encapsulation was performed using the methodology mentioned in Section 2.3.3, but only the chosen encapsulation matrices for each extract were utilized (MD for litchi and WP for coffee). Furthermore, it was carried out with the nine experimental conditions outlined in Table 2.

#### 2.4.3. Encapsulation Efficiency Based on Procyanidin Content

To measure the procyanidin content of the encapsulates, the process outlined in Section 2.3.4 was followed to release the procyanidins. The HCl–Butanol technique was then used to quantify these procyanidins, according to the previously described method. The encapsulation efficiency calculation relied on the amount of procyanidins measured in both the extract added to the microencapsulates and the procyanidins that were trapped in the systems.

#### 2.4.4. Total Polyphenols in Microcapsules

The total polyphenols were determined as described by [17] with some modifications. From the previously disintegrated microcapsules, 125 µL of sample was taken and placed in test tubes containing 50 µL of distilled water and 125 µL of the Folin–Ciocalteu reagent. Then, the tubes were stored in a dark location to prevent light exposure and allowed to settle for six minutes at room temperature. Next, 1250 µL of 7% Na_2_CO_3_ and 1000 µL of distilled water were added. The tubes were left for another 90 min at room temperature. Afterward, the absorbance of the samples was measured at 760 nm using a spectrophotometer, with water used as the blank. The absorbances were compared with a standard curve generated with gallic acid standard solutions (0–100 mg/L). The data are expressed as the gallic acid equivalent (GAE).

### 2.5. Evaluation of Antioxidant Activity

The antioxidant capacity of the extracts and encapsulates obtained during the experimental validation was assessed. The encapsulates underwent a release technique prior to analysis. Lipoperoxidation and ABTS methods were conducted using 10 mg of encapsulates in 1 mL of ethanol, while 15 mg in 0.5 mL water was used for DPPH.

#### 2.5.1. ABTS

An ABTS radical inhibition assay was performed in accordance with the methodology proposed in [18] with minor modifications. ABTS radical cation was generated from ABTS aqueous solution (7 mM) with potassium persulfate (2.45 mM) in the dark and at room temperature 12 h before use. Diluted solutions of ABTS were prepared in ethanol to an absorbance value of 0.700 ± 0.002 nm. Varying concentrations of the extracts and/or microencapsulates (50 µL) were allowed to react with 950 µL of the ABTS solution. After 1 min of reaction, the absorbance was measured at a wavelength of 734 nm. The ability to inhibit the radical (expressed as the percentage inhibition of the ABTS radical) was compared with gallic acid as a standard and calculated according to Equation (2):

(2) $$Inhibition\;\left(\%\right)=\frac{A-B}{A}\times 100$$

where *A* is the absorbance of the control reaction (containing all reagents, except for the test compound), and *B* sample is the absorbance with the test compound. The gallic acid equivalent (GAE/mL) was calculated with the slope equation obtained with the calibration curve.

#### 2.5.2. DPPH

To evaluate the free radical scavenging capacity of the extracts and encapsulates, the degree of discoloration caused by their components to a methanolic solution of DPPH was determined using the method of [18] with some modifications. First, the calibration curve was performed with solutions of gallic acid at concentrations of 2000, 1000, 500, 250, 125, 62.5, 31.25, 15.62, and 7.8 μg/mL. From which, 100 µL was taken in triplicate and placed in a test tube with 2.9 mL of a 200 µM DPPH solution prepared in methanol and allowed to stand for 30 min in the darkness. The absorbance of the mixture was measured spectrophotometrically at 517 nm. For the samples, 25 µL of sample was taken in triplicate and placed in test tubes with 725 µL of the DPPH solution, and the same procedure was followed. The radical scavenging capacity was calculated by means of Equation (2).

#### 2.5.3. Lipid Oxidation Inhibition (LOI)

To quantify the ability of the extracts and encapsulates to inhibit the generation of hydroxylated peroxides in the early stages of linoleic acid oxidation, the method described in [18] was used with minor modifications. The reaction was performed by placing 50 µL of the sample in a test tube with 100 µL of a gallic acid solution (0.6 g linoleic acid and 1.5 g Tween 20 in 8 mL ethanol) followed by the addition of 1500 µL of 0.02 M acetate buffer solution, pH 4. The mixture was homogenized and incubated at 37 °C for 1 min. Then, 750 µL of 2.5 M FeCl_2_ (0.01 g of FeCl_2_ and 0.017 g of EDTA in 100 mL of distilled water) was added, and 250 µL of each reaction was taken to measure the absorbance at time zero. The remaining solution was incubated at 37 °C for 24 h. After the incubation time, 250 µL aliquots were taken and transferred to test tubes with 1 mL of a 0.1 M NaOH solution (prepared in 10% ethanol) to stop the oxidation process and 2.5 mL of 10% ethanol to dilute the sample. The absorbance of the mixture was measured spectrophotometrically at 232 nm, using 10% ethanol as a blank and a mixture of the reagents without the sample as a control. The percentage inhibition of linoleic acid oxidation was calculated with Equation (3):

(3) $$Inhibition\;of\;lipid\;oxidation\;\left(\%\right)=\frac{A-B}{A}\times 100$$

where *A* is the difference between the absorbance of the control after 24 h and 0 h incubation, and *B* is the difference between the absorbance of each extract sample after 24 h and 0 h incubation.

#### 2.5.4. Morphological Characterization of the Microcapsules

The morphological characteristics of the microcapsules containing the polyphenols were identified using scanning electron microscopy (SEM) with an JEOL JSM-6610LV microscope (JEOL Inc., USA) with an acceleration voltage of 10 kV. Before imaging, each sample was sputter-coated with gold into a JEOL JFC-1100 sputter for 3 min. All the samples were processed and visualized at room temperature (~20 °C). Digital images were captured using Quartz PCI imaging software v8 (Quartz Imaging Corp., Vancouver, BC, Canada).

### 2.6. Statistical Analysis

The results are presented as mean ± standard deviation (SD). The data were analyzed using Statistica 10 software (Stat Soft, Tulsa, OK, USA). To determine the optimal conditions, the Taguchi method, the Tukey model for the selection of the encapsulation matrix, and T-student for the evaluation of antioxidant activity were used.

To experimentally validate the Taguchi methodology, we conducted encapsulations utilizing the optimal conditions obtained. The resulting microcapsules were subsequently used to assess antioxidant activities, procyanidin content, and morphology.

## 3. Results

### 3.1. Selection of the Encapsulation Matrix

#### 3.1.1. Procyanidin Content of Extracts

The result of the concentration of procyanidins in the extracts can be seen in Table S1. In the litchi residues, 12.13 mg of procyanidins per gram of dry matter was quantified. This represents 1.21% of the content in dry material. The coffee pulp had a lower content (6.66 mg of procyanidins per gram of dry material) than the litchi peel. The procyanidin content represented 0.66% of the dried by-product.

#### 3.1.2. Encapsulation Efficiency of Each Matrix

**Figure 1:**
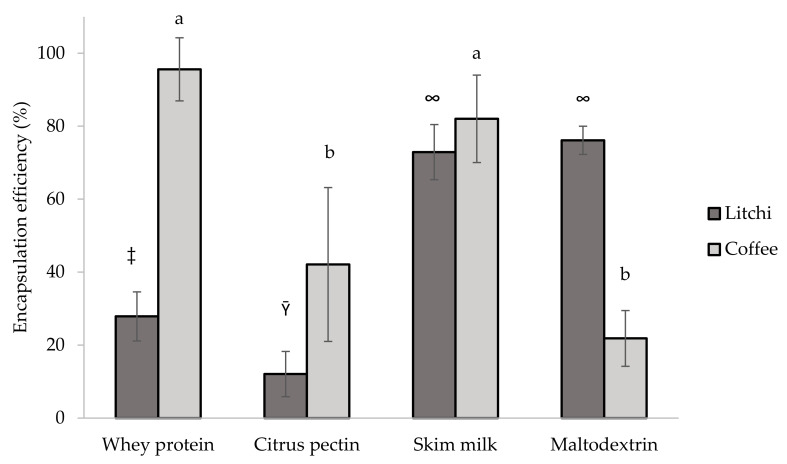
Procyanidin encapsulation efficiency of coffee pulp and litchi peel extracts using different polymers. Different letters and different symbols indicate significant difference (Tukey test, *p* ˃ 0.05).

After encapsulating the litchi and coffee pulp extracts using MD and SD as matrices, we evaluated the encapsulation efficiency (EE). The results are shown in Figure 1. MD (76.12 ± 3.86%) and SM (72.89 ± 7.55%) in combination with the litchi extract produced the highest values for encapsulation efficiency (EE). When it comes to the encapsulated coffee extract, the highest EE was achieved by using WP (95.57 ± 8.65) and SM (82.01 ± 7.65), as depicted in Figure 1.

### 3.2. Optimization of the Encapsulation Process

#### 3.2.1. Encapsulation Efficiency of Procyanidins and Total Polyphenols

The percentages of procyanidins and total polyphenols in the litchi microencapsulates for each of the nine treatments are presented in Table 3. For the content of procyanidins, treatments L1 and L3 yielded the lowest EE values at 59.9% and 62.1%, respectively. The highest EE values ranged from 84.3% to 98.0%. However, the quantification of total polyphenols resulted in lower EE values, ranging from 33.8% to 47.6%.

#### 3.2.2. Encapsulation Efficiency of Procyanidins and Total Polyphenols of Coffee Encapsulates

The experimental results for the coffee extracts’ EE are presented in Table 4. The higher-is-better category was applied to analyze the results, aiming to determine the best encapsulation conditions and maximize the EE of the microencapsulated bioactive compounds. The procyanidins’ EE values ranged from 77.2% to 93.5%, with the highest EE (93.5%) observed for treatment 6. The total polyphenol content was encapsulated at 239.0 ± 11.9 mg GAE/L, representing 93.3% of EE in treatment 5.

#### 3.2.3. Taguchi Analysis

##### Relative Influence of Encapsulation Factors

The impact of each factor involved in the encapsulation process of the two extracts is detailed in Table 5. The contribution or influence percentage of each factor involved in the potting process is crucial in determining the impact of modifying each factor on the EE values.

The contribution of each factor was analyzed using ANOVA and is expressed as a percentage (%). For the litchi (Table 5), it was evident that the feed flow had the highest percentage of contribution at 53.45%. This parameter, which controls the rate at which the sample enters the drying equipment, is significant since it determines the contact time of the sample with the other drying conditions. If the flow rate is excessively slow, the significance of the temperature and the exposure to air may harm the particle stability or bioactivity of the compound. Conversely, feeding the system at a rapid pace may cause inefficient drying and result in higher moisture values within the particles produced.

For the coffee (Table 5), the factor with the greatest contribution percentage was found to be the feed flow at 45.44%. However, a comparison of the contribution percentages of the other factors revealed that error made a significantly larger contribution (36%) than it did to the litchi and a significantly larger contribution than the other factors.

##### Individual Performance of Encapsulation Factors

**Figure 2:**
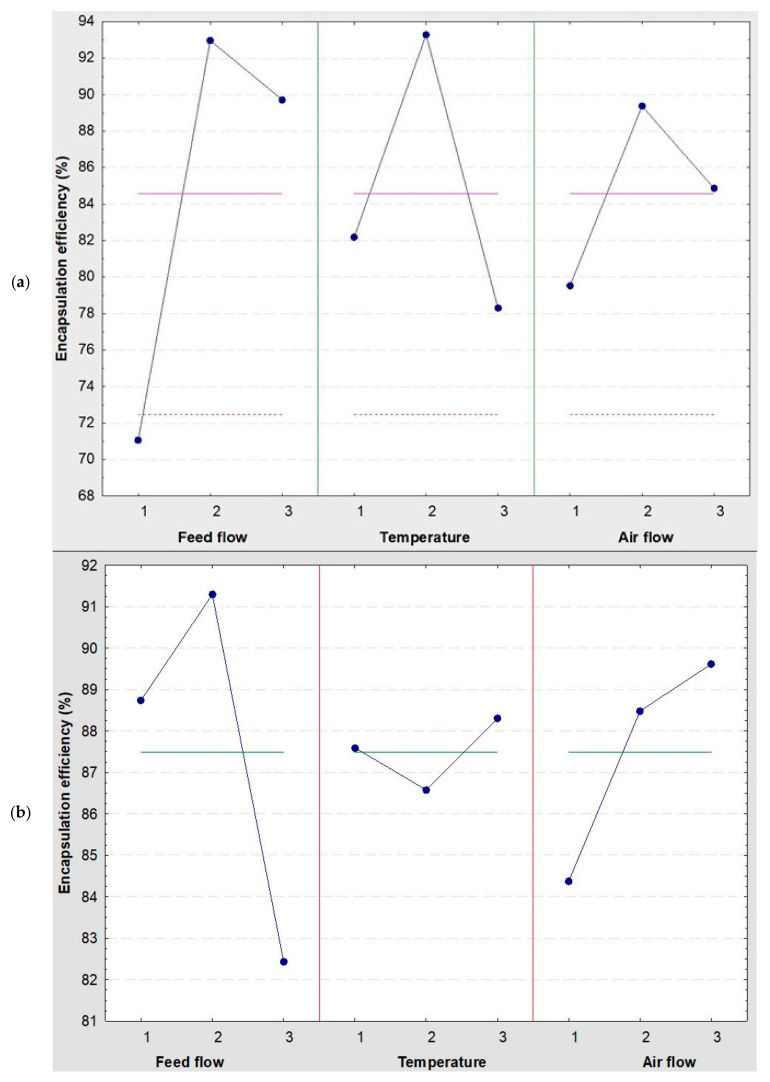
Individual performance of encapsulation factors at different levels. (**a**) Encapsulation of litchi extracts and (**b**) encapsulation of coffee extracts.

The diagram in Figure 2 depicts the distinct impact of each variable on the EE of the litchi and coffee extracts. In the litchi encapsulation results (Figure 2a), the feed flow displays no significant decrease at level 3 but a significant decrease at level 1. Meanwhile, temperature and airflow experience notable decreases at levels 1 and 3. The results for coffee encapsulation (Figure 2b) match those seen in litchi encapsulation, with airflow yielding superior outcomes under medium control. However, variables such as airflow and temperature demand higher settings to produce better results.

##### Optimal Encapsulation Conditions

The experiment’s results were analyzed using the highest-is-better category to determine the optimal encapsulation conditions for litchi and coffee extracts. Table 6 provides the details. For the litchi microcapsules, the highest encapsulation efficiency (EE) was achieved at a feed flow rate of 4.5 mL/min, a temperature of 150 °C, and an airflow rate of 35 m^3^/h (see Table 6). However, we adjusted the feed flow to 6 mL/min to shorten the process time while maintaining a high EE value (as shown in Table 6). As for the coffee microcapsules, the highest predicted EE value was achieved using a feed flow rate of 4.5 mL/min, a temperature of 175 °C, and an airflow rate of 40 m^3^/h (refer to Table 6), with a predicted value of 94.20%.

##### Experimental Validation of Optimum Encapsulation Conditions

The experimental validation conducted under the established optimal conditions for both extracts (Table 6) yielded EEs of 98.1% and 93.6% for the litchi and coffee, respectively. Based on the obtained and predicted values, the bias values for the litchi and coffee microcapsules (calculated using the formula mentioned in Table S2) were found to be 5.19% and 0.66%, respectively.

### 3.3. Evaluation of Antioxidant Activity

Table 7 presents the antioxidant capacity of the extracts and microcapsules derived from the litchi peel and coffee pulp. The results show a decrease in the antioxidant capacity of the microencapsulates. The LOI values of the microcapsules were 67.63 ± 5.31 and 55.28 ± 4.76 for the litchi and coffee microcapsules, respectively, indicating reductions of 16% and 30% in bioactivity compared to the extracts. The DPPH evaluation showed a similar pattern, with the bioactivity values of the litchi peel and coffee pulp remaining at 96.1% and 93.5%, respectively. The litchi peel extract demonstrated higher results in the ABTS assay (98.28 ± 0.24 GAE mg/mL). There were no statistical differences in the bioactivity of the coffee pulp extract and the microcapsules.

#### Microcapsule Morphology

**Figure 3:**
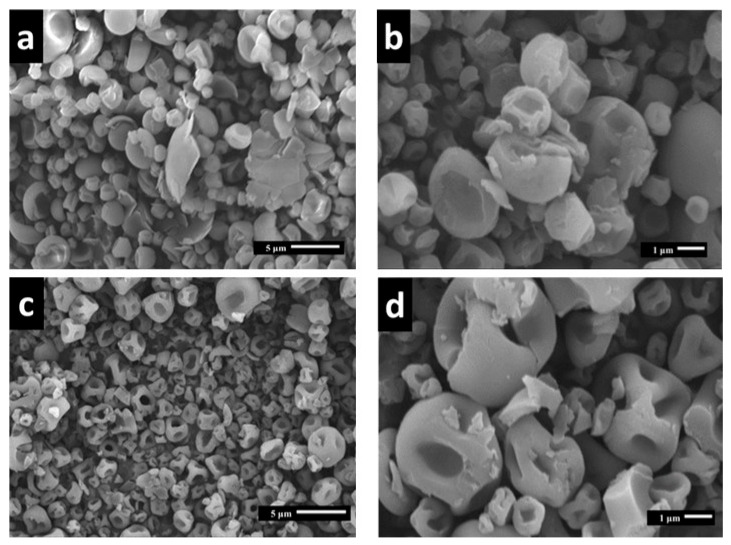
Scanning electron images of samples of microcapsules containing litchi procyanidins (**a**,**b**) and coffee procyanidins (**c**,**d**).

The SEM images in Figure 3 demonstrate that all microparticles produced with maltodextrin containing litchi (Lpro) had a spherical geometry but varied in terms of their surface morphology. The Lpro specimens (Figure 3a) exhibited primarily smooth surfaces, with certain specimens having less roughness than the coffee procyanidins (Cpro) (Figure 3b). However, the Cpro microparticles (Figure 3c,d) exhibited a strong invagination and rough texture. The Cpro microparticles had a higher mean particle diameter of 3.2 μm ± 1.4 μm than the Lpro microparticles, which had a mean diameter of 2.6 μm ± 1.1 μm.

## 4. Discussion

### 4.1. Procyanidins in Extracts and Encapsulation Matrix Selection

There are numerous studies mentioning the presence of procyanidins in various materials such as litchi and coffee, but their concentrations vary significantly. A study reported a procyanidin level of 2.2% (*v*/*v*) in the crude methanolic extract of litchi peel [19]. The procyanidin values in that study are slightly lower than those in the present work, but the extraction and quantification methods are different. However, the tannin concentrations in coffee pulp range from 0.23% [20], which is lower than the 2.01% (or 20.1 g/kg) of proanthocyanidins determined in this study. It should be noted that procyanidins, which also include prodelphinidins and propelargonidins [21], are directly derived from the aforementioned group.

The obtained particles, encapsulated with various matrices, exhibited significant variations in the preservation of bioactive compounds. It has been reported that MD is more suitable for encapsulating these compounds. It effectively preserves the functional properties of the encapsulated sample and produces capsules with more regular structures, enabling the components to remain within the wall material [22]. WP can produce small, stable capsules. They have circular shapes and sleek walls without any fissures and ensure gas safety [23].

The results of the spray-dried litchi extract particles, which exhibited higher encapsulation efficiency with MD, align with those of prior research indicating that MD is a potent encapsulating agent for this type of compound [22,24]. Additionally, previous studies have indicated the potential for phenolic compounds to form strong complexes with polysaccharides due to their water solubility, molecular size, conformational mobility, and polyphenol structure [25]. However, using MD for encapsulating coffee extract resulted in the lowest EE percentages. However, PS encapsulation resulted in the highest values. This suggests that there may be different types of procyanidin structures in coffee. These structures likely have higher degrees of polymerization than those found in litchi. Research has pointed out that the interaction between proteins and procyanidins increases with the degree of polymerization of procyanidins and the protein concentration [25,26,27,28]. Therefore, it is possible that coffee procyanidin structures have a higher degree of polymerization than those found in litchi. However, it has been reportedthat coffee pulp contains procyanidins with tetramer and pentamer structures [29], while [3,30] identified minor procyanidin structures, mainly dimers and trimers, in litchi. Furthermore, research has shown that the interaction between procyanidins and proteins increases with the degree of polymerization of the procyanidin. However, in this study, SM demonstrated good qualities as an encapsulating material for both extracts, resulting in satisfactory EE outcomes. However, MD and WP still exhibited superior EEs, which agrees with research [12] indicating contrasting impacts of several encapsulants for polyphenolic compounds in spray drying, with the most efficient encapsulant being MD as compared to SM and WP. The preceding phenomenon primarily resulted from the decreased stability of the microparticles produced by SM due to the capsules’ irregular and rough surface morphology [12]. It has been reported that these issues may occur due to the impact of the spray-drying conditions on the casein and the pH of the extract employed [31].

The efficient non-covalent binding between polyphenolic compounds such as proanthocyanidins and both carbohydrates and protein is attributed to hydrogen bonding and hydrophobic group interactions [12,26].

### 4.2. Optimization of Procyanidin Encapsulation

For the litchi extracts, the experimental results demonstrated a maximum procyanidin EE value of 98%. According to previous research reports, the EE of procyanidins using MD as the matrix ranges from 92.2% [32] to 79.3% [33]. This variation can be attributed to the dependence of encapsulation efficiency on the ratio between the encapsulated compounds and the matrix [34].

Despite this, the results obtained for the EE of polyphenols from litchi are consistent with those reported by [35], which encapsulated total polyphenols using microwave drying and spray drying and reported yields between 33.78% and 44.72%. However, previous studies have reported conservation rates of up to approximately 60% for polyphenols during the microencapsulation of cinnamon infusions under similar conditions. However, these studies did not incorporate optimization techniques into the encapsulation process [28].

The analysis examined how individual factors and their varying levels affected the microencapsulation of procyanidins from the litchi peel extracts. It was found that the feed flow had the greatest influence, accounting for 53.45% of the contribution. Level 2 resulted in the most favorable outcomes, followed by levels 1 and 3. At the lowest level, the sample flow is slow, which can cause damage to bioactive molecules due to high temperatures. Research has shown that compounds like polyphenols and procyanidins are particularly vulnerable to degradation over extended periods at high temperatures [36]. Therefore, temperature control is a crucial aspect to consider during the spray-drying process. The temperature at level 1 is too low to fully remove the water content of the wall material. This may lead to a decreased evaporation rate, deformation of the microcapsules, reduced fluidity, and potential agglomerations [37], ultimately decreasing performance [35]. The high final moisture percentage in the microcapsules hinders the quantification process of procyanidins. However, the decline in EE observed at the highest temperature can be attributed to the instability of procyanidins at extremely high temperatures. Additionally, it can result in excessive evaporation, membrane rupture, premature release, and degradation of the encapsulated ingredient. Similarly, a low volume of airflow can result in inadequate exposure of the wall material to air, which hinders effective water removal [37].

For the extracts obtained from the coffee pulp, the EE values exceed those obtained by [23]. They obtained an EE of 71.29% using WP and spray drying. The present work’s results surpass even those reported by [38], as they only achieved an EE of 50% under the same conditions. The contribution of the error term to the process was 36.33%, as shown in the residual (Table 5). This issue could potentially hinder the reproducibility of experiments. However, the Taguchi methodology suggests that this factor may not entirely signify an error in the process. It can also be attributed to the influence of two types of factors: factors not included in the experiment and uncontrollable factors, known as noise factors [39]. Therefore, it could be considered an error factor in the structure of the wall material and its properties. (No changes necessary.) Also, uncontrollable factors like ambient humidity and temperature have a direct impact on the sample temperature as it enters the drying equipment, as well as the emulsion and its ability to be uniformly sprayed. As the temperature of the feed increases, the viscosity and size of droplets may decrease. However, at high temperatures, some heat-sensitive ingredients may undergo volatilization or degradation [37].

The optimal performance of the three factors differed significantly from that in the litchi microencapsulation process. The microencapsulation of the procyanidins extracted from the coffee pulp through spray drying necessitated a feed flow of level 2 (4.5 mL/min), which aligns with the process utilized for litchi microencapsulation. However, higher levels were required for the temperature and airflow. This may be primarily attributed to the composition of the wall material. WP requires higher temperatures than MD for effective water removal from its structure. The optimal operating temperature for spray drying depends on the coating material and the type of phenolic compounds [12,24]. This can also explain the significant decrease in EE. When the feed rate is set to maximum, the sample flow becomes too rapid, resulting in a similar outcome to a reduced temperature. The greatest reduction in EE was observed at level 1 in regard to airflow. This led to inadequate drying of the sample, despite the system being set to the optimal temperature. Heat transfer occurs between the air and the product due to the temperature difference, resulting in the removal of water from the sample [37].

The results from the experimental validations of both extracts exceeded all values of EE achieved with the nine treatments of the experimental matrix. Although the predicted values were not reached, a significant decrease in the SD values was observed. This suggests that the optimal conditions obtained through the Taguchi methodology are highly reproducible. The experimental validation of both extracts is in good agreement with the expected data, showing a bias of less than 15% [39,40].

### 4.3. Microencapsulates Antioxidant Capacity and Morphology

Reactions involving antioxidant activity are intricately complex. Thus, to evaluate them under varying methods [18,41], it is crucial to evaluate the antioxidant capacity of encapsulated and non-encapsulated extracts. In the present study, the free radical scavenging capacity and the inhibitory effects on lipid oxidation were measured using three methods.

According to the findings, this study shows that the extracts have a significant capacity to generate hydrogen atoms as measured using the DPPH technique [42] and that microencapsulation efficiently preserves this property (96.1% and 93.5%, respectively, for the litchi rind and coffee pulp extracts). The litchi microcapsules demonstrated a reduced ability to yield only electrons measured using ABTS, while no significant differences were observed in the coffee extract and microencapsulates. The LOI activity decreased by 16% and 30% in the litchi and coffee microencapsulates, respectively.

This phenomenon may be explained by the fact that these properties are specific to various phenolic compounds, which tend to lose more during encapsulation than those having only an electron-donating ability [18]. The Tukey test enabled us to observe significant differences in antioxidant capacity between the extracts and microcapsules. However, it was determined that these properties can be adequately preserved through the encapsulation process. The extracts’ ability to inhibit lipid oxidation and their gallic acid equivalence remained intact, as none of the tests demonstrated a significant decrease in antioxidant activity between the extracts and the encapsulated ones.

The SEM analysis enabled us to ascertain the morphology and size of the microcapsules. The particles exhibited spherical shapes with diameters ranging from 2.6 to 3.2 μm and varied surface characteristics, contingent on the wall material. Previous studies have indicated that applying the spray-drying technique to encapsulate polyphenols coated with maltodextrin or a combination of maltodextrin and proteins resulted in the formation of microcapsules with rough surfaces and indentations [22].

## 5. Conclusions

This study explains the use of various encapsulation matrices for procyanidin-rich extracts obtained from litchi peel and coffee pulp. The litchi extract exhibited the highest encapsulation efficiency when the MD matrix was used, while the highest efficiency for the coffee extract was observed using the WP matrix. Through the utilization of the Taguchi experimental design, we optimized the process of encapsulation. The litchi extract showed the highest encapsulation efficiency under the conditions of a feed flow rate of 4.5 mL/min, a temperature of 150 °C, and an airflow of 35 m^3^/h. The ideal encapsulation parameters for the coffee extract were a feed flow rate of 4.5 mL/min, a temperature of 175 °C, and an airflow rate of 40 m^3^/h. The encapsulation efficiencies of procyanidins were 98.1% for the litchi and 93.6% for the coffee. The microencapsulation process effectively preserved the antioxidant activity of the procyanidins acquired from the extracts of the litchi peel and coffee pulp. The microcapsules had a particle range of 2.6 to 3.2 μm. This study indicates that the phenolic compounds present in the extracts could be suitable options as antioxidant agents. Moreover, the encapsulation method proved to be viable for maintaining such properties. The developed procyanidin encapsulations could serve as appropriate ingredients for incorporation into pharmaceutical or food products.

## Figures and Tables

**Table 1:** Parameters and levels assigned for the spray-drying encapsulation process.

Parameter	Level 1	Level 2	Level 3
Feed flow (mL/min)	3	4.5	6
Air inlet temperature (°C)	125	150	175
Drying airflow (m^3^/h)	30	35	40

**Table 2:** Experimental matrix for the orthogonal array L9 (3^3^).

Run No.	Feed Flow	Temperature	Airflow
1	1	1	1
2	1	2	2
3	1	3	3
4	2	1	2
5	2	2	3
6	2	3	1
7	3	1	3
8	3	2	1
9	3	3	2

**Table 3:** Encapsulation efficiency (EE) and concentrations of procyanidins and total polyphenols in the litchi microencapsulates.

	Procyanidins *		Total Polyphenols *	
Run	Concentration (mg/L)	EE (%)	Concentration (GAE mg/L)	EE (%)
L1	57.1 ± 23.0	59.6 ± 24.0	112.8 ± 32.5	38.6 ± 11.1
L2	87.7 ± 14.4	87.0 ± 15.1	98.7 ± 18.0	33.8 ± 6.2
L3	59.5 ± 11.0	62.1 ± 11.5	112.3 ± 10.5	38.5 ± 3.6
L4	88.5 ± 0.5	92.3 ± 0.5	112.3 ± 14.6	38.5 ± 5.0
L5	98.3 ± 2.3	98.0 ± 2.3	131.3 ± 11.3	45.0 ± 3.9
L6	88.8 ± 8.7	88.6 ± 8.7	139.0 ± 6.1	47.6 ± 6.1
L7	94.8 ± 3.7	94.6 ± 3.6	129.5 ± 5.1	44.3 ± 1.8
L8	90.6 ± 6.3	90.3 ± 6.2	115.3 ± 26.9	39.5 ± 9.2
L9	84.5 ± 14.9	84.3 ± 14.8	121.5 ± 22.0	41.6 ± 7.5

* The means and standard deviation of the results are presented. Only the EE results of the procyanidins of both extracts were analyzed with the Taguchi method.

**Table 4:** Encapsulation efficiency (EE) and concentrations of procyanidins and total polyphenols in coffee encapsulations.

	Procyanidins *		Total Polyphenols *	
Run	Concentration (mg/L)	EE (%)	Concentration (GAE mg/L)	EE (%)
L1	55.0 ± 1.2	82.4 ± 1.7	202.5 ± 3.7	62.2 ± 15.3
L2	62.4 ± 4.4	93.4 ± 6.5	206.9 ± 22.6	72.1 ± 7.9
L3	60.4 ± 3.5	90.5 ± 5.3	196.2 ± 13.5	68.4 ± 4.7
L4	60.9 ± 1.9	91.2 ± 2.8	226.0 ± 19.3	78.8 ± 6.7
L5	59.6 ± 3.9	89.2 ± 5.9	239.0 ± 11.9	83.3 ± 4.1
L6	62.5 ± 6.2	93.5 ± 9.3	227.3 ± 14.6	79.2 ± 5.1
L7	59.6 ± 2.8	89.2 ± 4.2	216.6 ± 15.0	75.5 ± 5.2
L8	51.6 ± 1.6	77.2 ± 2.4	230.5 ± 26.0	80.4 ± 9.1
L9	54.0 ± 4.5	80.9 ± 6.7	214.2 ± 22.7	74.7 ± 7.9

* The means and standard deviation of the results are presented. Only the EE results of the procyanidins of both extracts were analyzed with the Taguchi method.

**Table 5:** Relative influence of the factors in the encapsulation process of litchi and coffee extracts.

Extract	Factors	SS	df	MS	F	p	Contribution (%)
Litchi	Feed flow	836.91	2	418.45	3.81	0.20	53.45
	Temperature	363.16	2	181.58	1.65	0.37	23.19
	Airflow	146.03	2	73.01	0.66	0.60	9.33
	Residual	219.59	2	109.79			14.03
	Total	1565.71					100
Coffee	Feed flow	124.68	2	62.34	1.25	0.44	45.44
	Temperature	4.47	2	2.23	0.04	0.95	1.63
	Airflow	45.54	2	22.77	0.45	0.68	16.60
	Residual	99.67	2	49.83			36.33
	Total	274.38					100

**Table 6:** Optimal encapsulation conditions for procyanidins obtained from litchi extracts a)-b), and coffee extracts c).

Extract	Factors	Level	Value	Standard Error
Litchi	Feed flow	2	4.5 mL/min	6.05
	Temperature	2	150 °C	6.05
	Airflow	2	35 m^3^/h	6.05
	Predicted EE	106.45%		
Litchi-adjusted	Feed flow	3	6 mL/min	6.05
	Temperature	2	150 °C	6.05
	Airflow	2	35 m^3^/h	6.05
	Predicted EE	103.20%		
	Experimental EE	98.10%		
Coffee	Feed flow	2	4.5 mL/min	4.07
	Temperature	3	175 °C	4.07
	Airflow	3	40 m^3^/h	4.07
	Predicted EE	94.20%		
	Experimental EE	93.60%		

**Table 7:** Antioxidant capacity of extracts and microcapsules.

Sample	LOI (%)	DPPH (GAE μg/mL)	ABTS (GAE μg/mL)
Litchi extract	80.50 ± 1.28 ^a^	88.15 ± 0.16 ^a^	98.28 ± 0.24 ^a^
Coffee extract	78.58 ± 2.70 ^a^	88.06 ± 0.54 ^a^	93.10 ± 0.40 ^b^
Litchi microcapsules	67.63 ± 5.31 ^b^	84.75 ± 0.76 ^b^	93.14 ± 0.79 ^b^
Coffee microcapsules	55.28 ± 4.76 ^c^	82.38 ± 0.39 ^c^	93.14 ± 0.92 ^b^

LOI = lipid oxidation inhibition; GAE = gallic acid equivalent. Similar letters in each column indicates that the samples are statically equal (Tukey test *p* ≤ 0.05).

## Data Availability

The datasets used in the current study are available from the corresponding author on reasonable request.

## Supplementary Materials

The following supporting information can be downloaded at: https://www.mdpi.com/article/10.3390/polym15183823/s1, Table S1. Procyanidin concentration in litchi and coffee samples. Table S2. Predicted and experimental values of the percentage of EE for litche and coffee extracts.
