# Supplementary material for: The Specific Encapsulation of Procyanidins from Litchi Peel and Coffee Pulp Extracts via Spray-Drying Using Green Polymers
Source: Polymers (Basel). 2023 Sep 19;15(18):3823. doi: 10.3390/polym15183823 (PMC10537477; doi:10.3390/polym15183823)
Supplement: Supplementary file 1 [file polymers-15-03823-s001.zip › polymers-2562859-supplementary.pdf]

Article

The Specific Encapsulation of Procyanidins from Litchi Peel and Coffee Pulp Extracts by Spray-Drying using Green Polymers

María de los Ángeles Vázquez-Núñez [1,†], Mayra Aguilar-Zárate [2,†], Ricardo Gómez-García [3], Carlos Reyes-Luna [4], Pedro Aguilar-Zárate [4,*] and Mariela R. Michel [4,*]

[1] Facultad de Estudios Profesionales Zona Huasteca, Universidad Autónoma de San Luis Potosí, Romualdo del Campo 501, Colonia Rafael Curiel, Ciudad Valles, San Luis Potosí, C. P. 79060, México
[2] Facultad de Ciencias Químicas-CIEP, Universidad Autónoma de San Luis Potosí, Av. Dr. Manuel Nava 6, Zona Universitaria, 78210 San Luis Potosí, México
[3] Universidade Católica Portuguesa, CBQF - Centro de Biotecnologia e Química Fina – Laboratório Associado, Escola Superior de Biotecnologia, Rua Diogo Botelho 1327, 4169-005 Porto, Portugal
[4] Engineering Department, Tecnológico, Nacional de México/I. T. de Ciudad Valles, Carretera al Ingenio Plan de Ayala Km 2, Colonia Vista Hermosa, 79019, Ciudad Valles, San Luis Potosí, México
* Correspondence: pedro.aguilar@tecvalles.mx (P.A.-Z.); mariela.michel@tecvalles.mx (M.R.M.)
† These authors contributed equally to this work.

1. Supplementary Material

Table S1. Procyanidin concentration in litchi and coffee samples.

Sample	PCs in Extract (mg/L)	mg of PCs/g dry Matter	Content (% w/w)
Litchi	1213.48 ± 11.4	12.13 ± 0.11	1.21
Coffee	665.68 ± 8.5	6.66 ± 0.08	0.66

PCs = Procyanidins.

Table S2. Predicted and experimental values of the percentage of EE for litche and coffee extracts.

	Predicted Value	Experimental Value	Bias *
Litchi	103.20	98.1 ±0.2	5.19
**Coffee**	94.22	93.6 ± 3.5	0.66

* Bias (%) = (predicted value – experimental value) /experimental value * 100
